# Evolution of blood-brain-barrier permeability after acute ischemic stroke

**DOI:** 10.1371/journal.pone.0171558

**Published:** 2017-02-16

**Authors:** Zamir Merali, Kun Huang, David Mikulis, Frank Silver, Andrea Kassner

**Affiliations:** 1 Department of Medical Imaging, University of Toronto, Toronto, Ontario, Canada; 2 Division of Physiology and Experimental Medicine, Hospital for Sick Children, Toronto, Ontario, Canada; 3 Department of Medicine, University of British Columbia, Vancouver, BC, Canada; 4 Division of Neuroradiology, Joint Department of Medical Imaging, Toronto Western Hospital, Toronto, Ontario, Canada; 5 Division of Neurology, Toronto Western Hospital, Toronto, Ontario, Canada; Hungarian Academy of Sciences, HUNGARY

## Abstract

The dynamics of BBB permeability after AIS in humans are not well understood. In the present study we measured the evolution of BBB permeability after AIS in humans using MRI. Patients presenting to our institution with a diagnosis of AIS underwent a single dynamic contrast-enhanced MRI (DCE-MRI) sequence to measure BBB permeability during their initial workup. Forty-two patients were included in the final analysis. The patient sample underwent DCE-MRI at a mean time of 23.8hrs after the onset of AIS symptoms (range: 1.3–90.7hrs). At all time-points the BBB permeability within the infarct region of the brain as defined on DWI/ADC was higher compared to the homologous region of the contralateral hemisphere (p<0.005). BBB permeability, expressed as a ratio of infarct permeability to contralateral permeability, was greatest at 6-48hrs after the onset of AIS. Although the data was not acquired longitudinally, these findings suggest that the permeability of the BBB is continually elevated following AIS, which contradicts previous assertions that BBB permeability after AIS follows a biphasic course. Knowledge of BBB dynamics following AIS may provide insight into future treatments for AIS, especially BBB stabilizing agents.

## Introduction

Blood-brain-barrier (BBB) degeneration after acute ischemic stroke (AIS) may lead to pathologic processes such as edema and hemorrhagic transformation (HT) [1–4]. These pathologic processes are associated with negative clinical outcomes through exacerbation of brain injury [3,5]. Although BBB degeneration is known to evolve with time after the onset of cerebral ischemia, very little is currently known about the magnitude and temporal evolution of the damage in humans after AIS. A more detailed understanding of BBB evolution after AIS would be important to guide future treatments for AIS such as BBB stabilizing and neuroprotective agents.

Studies in animal models have shown that BBB degeneration after cerebral ischemia-reperfusion may follow a biphasic course [6–8]. In this paradigm, an early increase in permeability is followed by a refractory period during which BBB permeability returns to baseline, and a delayed second increase in permeability. Recent studies, however, have cast doubt on this interpretation by showing incomplete recovery after the first increase in BBB permeability, which indicates a continuous leakage for days to weeks without any appreciable change in magnitude [9–14]. Unfortunately, longitudinal studies assessing BBB permeability after stroke to date have been limited to animal models. Many differences exist in the response to cerebral ischemia between animals and humans [15–17]. Therefore, a study to determine the magnitude and temporal evolution of BBB damage after AIS in humans would provide more clinically relevant information that could better guide future basic and clinical research. Such a study could be made possible through the use of advanced imaging techniques, such as dynamic contrast-enhanced magnetic resonance imaging (DCE-MRI), which provides a non-invasive approach to estimate BBB permeability by modeling contrast agent leakage through the BBB [18].

In the present study we attempted to measure and estimate the magnitude and temporal evolution of BBB permeability in humans with AIS. We hypothesized that, similarly to recent controlled animal studies using DCE-MRI, BBB permeability in humans would remain elevated after AIS instead of following a biphasic pattern.

## Materials and methods

### Study design

The study was a retrospective review of MRI data acquired from an earlier study performed at the Toronto Western Hospital between 2003 and 2006. In the previous study, patients presenting to the hospital with a working diagnosis of AIS based on clinical assessment and CT findings as well as a safe screening profile for MRI were imaged. To minimize the effect of confounding factors and co-morbidities on our measures, patients with non-stroke lesions shown on CT, prior history of intracranial hemorrhage, uncontrolled hypertension, seizure at onset of stroke, known bleeding diathesis, and abnormal blood glucose levels were excluded. All eligible patients underwent an MR scanning protocol that included DCE-MRI. One patient was found to meet criteria for thrombolytic therapy and received a recombinant tissue plasminogen activator (rt-PA) infusion during the MR protocol. However, this was only performed after the DCE-MRI scan was completed. Thus, thrombolysis did not influence the BBB permeability during the DCE-MRI scan.

For the current review, patient data were grouped by the time elapsing from the reported onset of stroke symptoms to time of DCE-MRI. The groups were as follows: hyperacute (<6hrs), acute (6-48hrs), subacute (>48hrs). In addition, we separated patients based on whether they later on proceeded to HT. This separation was necessary as previous work has shown that AIS patients who progressed to HT exhibit significantly higher BBB permeability than those who do not [19].

The review was performed in accordance with the institutional guidelines for human research and were approved by the University Health Network Research Ethics Board. All participating subjects (or their substitute decision-makers) provided written informed consent.

### MR imaging protocol

All subjects were imaged on a 1.5T clinical MR imaging system (Signa Excite; GE Healthcare), equipped with an 8-channel head coil. MR imaging consisted of a comprehensive acute stroke MR imaging protocol including anatomic, whole-brain perfusion- and diffusion-weighted imaging (DWI) of both hemispheres excluding the cerebellum, contrast-enhanced MR angiography, and high-resolution post-contrast T1-weighted imaging as described by Kassner et al [19]. In addition, a DCE-MRI sequence was performed to assess BBB permeability. The DCE-MRI scan was always obtained before both perfusion-weighted MR imaging and contrast-enhanced MR angiography. Note that these data were collected before the 2006 US Food and Drug Administration (FDA) public health advisory statement regarding nephrogenic systemic fibrosis. We, therefore, no longer use this high-dose protocol in patients with poor or uncertain renal status.

DCE-MRI parameters were as follows: dynamic 3D gradient echo, FOV = 240 mm, matrix = 128×128, slice thickness = 5 mm, TR = 5.9 ms, TE = 1.5 ms, FA = 20°, temporal resolution = 9 sec, volumes = 31. The total imaging time was 4:48 min. The DCE-MRI sequence covered the entire infarct in all cases. During the DCE-MRI scan each patient received a total dose of 15 mL of gadodiamide (Omniscan formulation; GE Healthcare, Milwaukee, Wis), which was injected at a rate of 5 mL/sec.

### Image analysis

Retrieved data were transferred to an independent workstation for image registration and further analysis. Diffusion-weighted images with b = 0, 1000 were converted to mean apparent diffusion coefficient (ADC) maps by using in-house software (MR analyst, Version 1.3; University of Toronto, Toronto, Ontario, Canada). ADCs for a given direction were calculated by fitting the normalized logarithmic signal-intensity decay as a function of the b-value. Areas of ischemia were identified as regions of reduced diffusion relative to normal cortex on ADC maps and were the basis for the lesion region-of-interest (ROI) selection. In addition, a second ROI within the homologous location in the contralateral hemisphere was defined to represent healthy tissue. The ROIs of the ADC maps were saved and later copied onto the DCE images for BBB permeability analysis.

Image registration was performed by using an automated local affinity model implemented in Matlab (Version 6.3, MathWorks, Natick, Mass) to maximize mutual information between datasets. Parametric maps of permeability-surface area product (KPS) were calculated on a pixel-by-pixel basis using an in-house image analysis tool (MR Analyst v 1.3) written in Matlab (Mathworks, Natick, Mass). The software utilizes the Patlak analysis, which is based on a unidirectional 2-compartment pharmacokinetic model to characterize the behavior of contrast agent leaking across the BBB [20]. KPS was calculated by relating the time-varying MR signals from the extravascular (tissue) region to an intravascular (input) function via the model and performing a linear regression to estimate the rate of leakage exhibited in a given region. The input function for all patients was obtained in the sagittal sinus, as it provides a reasonable surrogate of the arterial input function as discussed previously by Ewing et al [21]. The T1 value of blood was assumed in all stroke cases to be 800ms. Under the assumption that the arterial and capillary concentration of the contrast agent are sufficiently similar [22], the leakage was expressed as KPS with units of mL/100 g/min. In a separate analysis infarcted KPS values were normalized against the homologous normal tissue in the contralateral hemisphere. Thus a ratio of infarcted KPS to contralateral KPS (KPS_i/c_) was generated. Experimenters were blinded to group allocation during image analysis.

### Statistical analysis

In our primary analysis infarct KPS (KPS_i_) values were compared between the hyperacute, acute, and subacute groups of patients with a one-way ANOVA. The same test was applied to detect group differences in mean NIHSS and lesion volume. This was followed by a Tukey's Honest Significant Difference test to determine which groups in the sample differed. In addition, we performed a two-tailed paired Student's t-test between the infarct and contralateral KPS values within each group to demonstrate elevated leakage in the lesion area.

In our secondary analysis KPS was considered as a ratio of infarcted KPS to contralateral KPS (KPS_i/c_). In this way infarcted KPS was normalized against the normal tissue in the contralateral hemisphere to reduce variability due to patient specific tissue characteristics. The KPS_i/c_ was compared between the hyperacute, acute, and subacute groups of patients with a one-way ANOVA followed by a Tukey's Honest Significance Difference test. All statistical analyses were completed using RStudio (RStudio Team 2015, version 0.99.467).

## Results

We identified 49 AIS patient datasets with successful DCE-MRI scans. A total of 7 datasets were from patients who later proceeded to HT. As expected, the patients who proceeded to HT exhibited significantly higher KPS values those who did not (KPS_i_ = 1.44±0.76 vs 0.77±0.47 mL/100g/min, KPS_i/c_ = 2.77±1.29 vs 1.45±0.65). The data for the HT group were only acquired during the hyperacute phase, so analysis of the temporal differences in HT patients was not possible. Therefore, the remainder of the paper will focus on the data acquired from the 42 non-HT patients. General characteristics of the non-HT sample population can be seen in (Table 1).

**Table 1:** General demographic and clinical characteristics of the sample population.

Variable	*n = 42*
Male gender	57.1%
Age (yr)	59.4 (range: 28–94)
NIHSS	5.2 (range: 0–12)
Time between stroke onset and MRI (hr)	23.8 (range: 1.3–90.7)
Received tPA	19.0%
Stroke subtype:	
Cortical infarct	n = 23
Lacunar infarct	n = 5
Lenticulostriate artery GM infarct	n = 3
Lenticulostriate artery WM infarct	n = 4
Lenticulostriate artery GM + WM infarct	n = 1
Cortical + Lenticulostriate artery WM infarct	n = 2
Cortical + watershed infarct	n = 2
WM perforating artery infarct	n = 1
Brainstem	n = 1

**Fig 1:**
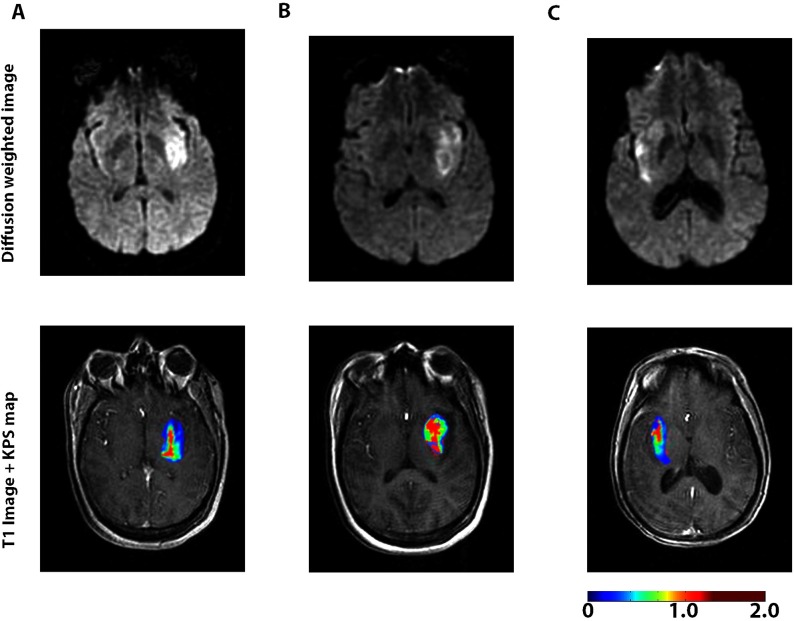
Representative diffusion-weighted scan (above) and blood-brain-barrier permeability map (below) for 3 patients. **These patients presented <6hrs (A), 6-48hrs (B), and >48hrs (C), following the onset of stroke symptoms.** The scale represents KPS in mL/100g/min.

Non-HT patients were grouped by the time since the reported onset of stroke symptoms. Group sizes were as follows: hyperacute phase (n = 20), acute phase (n = 11), and subacute phase (n = 11). Patients underwent DCE-MRI at an average of 23.8hrs after stroke onset (Range: 1.3–90.7hrs). Nineteen percent of patients received rt-PA after DCE-MRI was completed. At the time of the DCE-MRI scan all patients showed substantial infarcts on DWI (Fig 1), which did not significantly differ in volume between groups (p = 0.30). A visual inspection of KPS maps revealed that leakage tended to be higher within the core of the infarct and relatively less pronounced in the periphery of the infarct (Fig 1). The NIHSS, taken on admission to the emergency room, also did not significantly differ between groups (p = 0.31).

**Fig 2:**
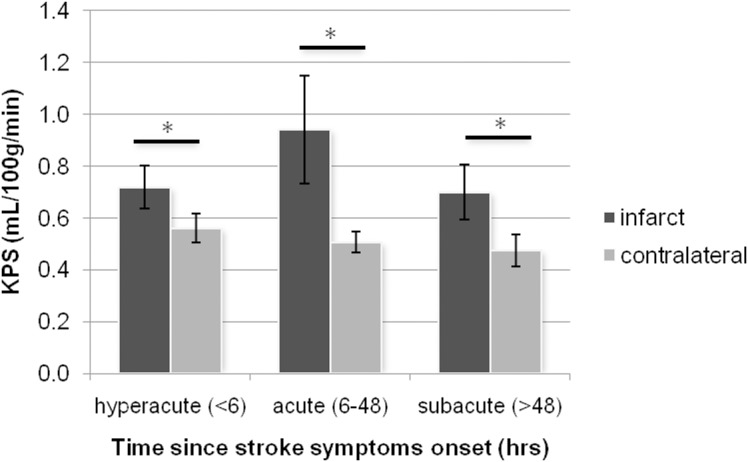
Blood-brain-barrier permeability within the infarct and a homologous region in the contralateral hemisphere stratified by time since stroke symptom onset. Error bars represent standard error of the mean. (*) p<0.05 two-tailed Students t-test.

KPS, ADC, and infarct size data for each group can be seen in Table 2. In all groups mean KPS values within the infarct were significantly higher than those measured in the contralateral hemisphere (p<0.005) (Fig 2).

**Table 2:** Permeability-surface area product (KPS) and apparent diffusion coefficient (ADC) values within the infarct and contralateral regions stratified by time since stroke symptom onset.

	hyperacute	acute	subacute
	(<6hrs)	(6-48hrs)	(>48hrs)
**infarct KPS (mL/100g/min)**	0.72 ± 0.37	0.94 ± 0.69	0.70 ± 0.35
**contralateral KPS (mL/100g/min)**	0.56 ± 0.26	0.51 ± 0.14	0.48 ± 0.20
**infarct ADC (×10**^**−4**^**)**	5.93 ± 1.49	6.42 ± 1.27	6.21 ± 1.76
**contralateral ADC (×10**^**−4**^**)**	8.52 ± 1.48	8.50 ± 1.07	8.70 ± 1.47
**Infarct volume (cm**^**3**^**)**	2.89 ± 2.82	2.27 ± 2.39	5.31 ± 8.38

Values expressed in mean ± standard deviation.

In the hyperacute group (<6hrs) KPS_i_ was 0.72 ± 0.37 mL/100g/min (Fig 2). In the acute group (6-48hrs) the KPS_i_ had risen to 0.94 ± 0.69 mL/100g/min, which was higher than the hyperacute group (p = 0.42). In the subacute group (>48hrs) the KPS_i_ had decreased to 0.70 ± 0.35 mL/100g/min (p = 0.45). However, these changes were not statistically significant.

**Fig 3:**
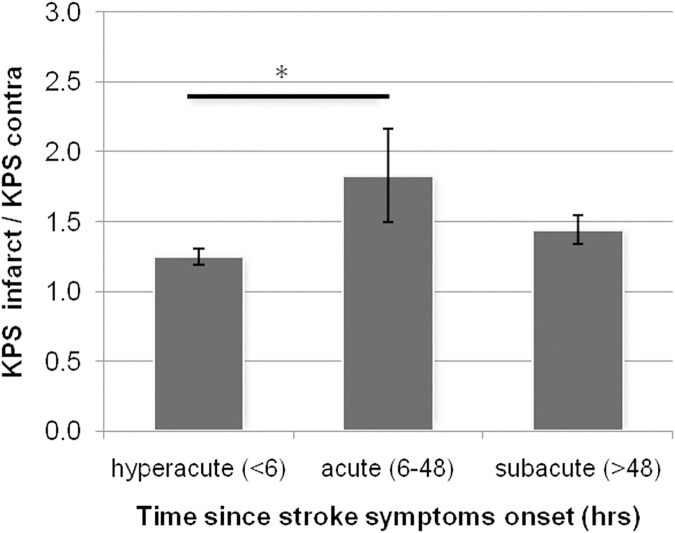
Infarct blood-brain-barrier (BBB) permeability normalized to contralateral BBB permeability stratified by time since stroke symptom onset. Error bars represent standard error of the mean. (*) p<0.05 Tukey's honest significant difference test.

In a secondary analysis, KPS was considered as a ratio of the contrast agent leakage measured in the infarcted ROI versus the contralateral ROI (KPS_i/c_) (Fig 3). In the hyperacute group the KPS_i/c_ was 1.25 ± 0.27. In the acute group the KPS_i/c_ had increased to 1.83 ± 1.11, which was significantly different to the hyperacute group (p<0.05). In the subacute group the KPS_i/c_ had reduced to 1.44 ± 0.33, which was not significantly different from the acute (p = 0.31) or hyperacute group (p = 0.68).

## Discussion

This study was a retrospective review of MRI data to investigate the temporal evolution of BBB permeability in humans after AIS. BBB permeability was assessed using DCE-MRI in 49 patients presenting with AIS to our facility. Seven patients were found to have proceeded to HT, and their data were separated from the non-HT group and not included in longitudinal analysis. For the 42 remaining non-HT patients, the primary finding of the present study was that a continuous BBB leakage could be confirmed with DCE-MRI in humans for up to 90.7 hrs after AIS. Thus, we did not observe a biphasic pattern of BBB disruption in our sample population.

The breakdown of the BBB is a complex process driven by a cascade of mediating factors including inflammatory processes, formation of oxygen reactive species, and activation of matrix metalloproteinase-9 (MMP-9) [2,16,23,24]. However, the extent to which each of these processes contributes to the degradation of the BBB remains an active area of research and the progression of BBB injury is still not fully understood. Animal studies examining BBB permeability after ischemia-reperfusion have produced conflicting results, with most suggesting a biphasic pattern while other more recent studies have found a continuously elevated permeability of the BBB for up to 1 week.

In animal models of ischemia-reperfusion, biphasic behavior of BBB disruption has referred to a pattern of ‘open-closed-open’ where a period of increased permeability is followed by a return to baseline and a second period of increase. However, articles that reported such a biphasic pattern of BBB permeability have differed in their reported time-point for BBB closure. The period of BBB closure has been reported to occur at 48hrs [7], 24hrs [6], 3hrs [25], and 1-4hrs [8]. It is worth noting that the methods of assessing BBB permeability and animal models have also differed greatly between these studies, which may account for inconsistencies in the reported pattern of BBB disruption. For example a tracer with small molecular weight may display different leakage characteristics from a larger tracer. A spontaneously hypertensive rat subjected to 2hrs of ischemia [8] may not be comparable to 1hr of ischemia in the cat [25]. More recent studies using tighter experimental controls have tended to contradict the prior finding of biphasic BBB opening and have instead suggested a gradual increase in BBB permeability during the first 24hrs after ischemia-reperfusion [9–14]. Few studies have measured BBB permeability beyond 24hrs, but available evidence suggests that the permeability of the BBB remains elevated up to 1 week [26].

In the present study we observed that KPS measured in the infarct core (KPS_i_) was elevated relative to the contralateral KPS (KPS_c_) at all time-points, suggesting a sustained increase in BBB permeability after AIS. Moreover, the magnitude of KPS_i_ exhibited a trend, in which the acute group had a higher leakage compared to either the hyperacute or subacute groups. After normalizing with KPS_c_, the acute group (6-48hrs) exhibited significantly elevated leakage suggesting higher BBB permeability compared to the hyperacute group. Pathologic cerebral edema, which is believed to result from increased BBB permeability, tends to develop within the first 24 to 48 hours after AIS [27]. In addition, the risk of hemorrhagic transformation, also believed to be associated with increased BBB permeability, is highest during the acute phase after AIS [28]. Thus, our observation of elevated BBB permeability during the acute phase after AIS correlates temporally with the established period during which complications of increased BBB permeability are most likely to develop. However, higher KPS variability was also observed in the acute phase, suggesting that the degree of BBB opening is not consistent across all subjects during this time period. This may be due to the wide time range of the acute phase, which was based on clinical definitions. While this period is generally associated with a higher risk of complications in AIS, it may be possible that the higher BBB permeability associated with these complications only exist within a smaller window within the acute phase. Alternatively, the variability may indicate that a subgroup of patients in our data do not adhere to the trend and follow a different pattern of BBB permeability. This effect will be the topic of future research as the delineation of subgroups is not feasible with the current sample size in our study.

Our findings are further supported by previous studies that performed qualitative assessment BBB integrity using post-contrast parenchymal enhancement on T1 weighted images [29–31]. These studies observed hyperintense regions due to contrast leakage through the BBB only after the hyperacute stage in acute ischemic stroke, suggesting that a significant change in permeability occurs during the acute phase. In a separate study investigating the effect of thrombolytic therapy, the administration of rtPA lead to an increase of parenchymal contrast enhancement during the hyperacute phase [32]. While the visual assessment of contrast leakage on structural imaging is a simpler approach, the enhancement can be too subtle to detect and its origins may be attributed to elevated fractional blood volume instead of contrast extravasation [33]. Thus, calculation of KPS remains the more robust and sensitive technique as it was able to show increased leakage in the hyperacute stage and provide quantifiable evidence of BBB disruption over time.

As this study is a retrospective analysis of AIS patients at our institution, we were not able to follow patients longitudinally. This is a limitation of our analysis, resulting in the grouping of our patients into hyperacute, acute, and subacute phases to study the time course of BBB disruption using group averages. This was unavoidable and the variability in stroke severity between patients within each group may dampen trends in the temporal pattern of BBB disruption. It is worth noting that infarct volume and NIHSS did not differ significantly within groups or between groups. Our retrospective study may guide future prospective study designs attempting to assess BBB dynamics after AIS.

A minor limitation of our study is the manual selection of ROIs. To reduce inter-observer variability, the same investigator placed all ROIs during the experiment. We also did not collect data beyond 100hrs of stroke onset, despite certain animal models suggesting that BBB permeability may remain elevated for at least 1 week after stroke onset. However, complications related to increased BBB permeability occur most often within the first 48hrs after stroke onset, which was captured in our study design. Also, the imaging protocol did not include a T1 mapping sequence due to the strict time constraints for the management of acute stroke patients. Without the characterization of T1 inhomogenieties across different tissues, errors may be introduced in the conversion from signal enhancement to contrast concentration and skew the KPS estimates [34]. Heye et al reported the use of variable flip angle to quantify T1 for DCE imaging in patients with mild ischaemic stroke approximately 1 month after first presentation [22]. However, in an acute clinical setting, the acquisition of a T1 map was not feasible at our institution. Lastly, modeling of BBB permeability necessitates certain assumptions about the cerebral circulation. Our analysis assumed that the contrast concentration in the input function is effectively equal to the concentration in the capillaries and that leakage of contrast through the BBB is unidirectional, which are plausible conditions in AIS [18]. Also, the term KPS is inherently associated with the capillary surface area, which can be affected by the ischemic injury caused by stroke. However, due to the retrospective nature of the study, our data does not provide any means to assess vessel density and size to accurately model the endothelial surface area. Nonetheless, previous results from KPS measures in AIS patients based on the assumptions used here have been shown to be clinically useful as they could detect permeability changes caused by rtPA administration and identify patients who would proceed to HT [19].

## Conclusion

The present study assessed the temporal evolution of BBB permeability in humans after AIS. The major finding of this study is that BBB permeability within infarcted tissue remained elevated at all time-points, which supports recent animal studies that have suggested a continually elevated BBB permeability after ischemic injury. A secondary finding is that BBB permeability may be most elevated at 6-48hrs after stroke onset. Detailed knowledge of BBB dynamics after AIS is important when considering future treatments for AIS such as BBB stabilizing and neuroprotective agents.

## Supporting information

S1 Table Recorded MRI and clinical measurements of each patient. (XLSX)
